# Frontline treatments in extremely elderly patients with diffuse large B-cell lymphoma: a population-based study in Taiwan, 2010–2015

**DOI:** 10.1186/s12979-020-00188-8

**Published:** 2020-06-10

**Authors:** Huai-Hsuan Huang, Bor-Sheng Ko, Ho-Min Chen, Li-Ju Chen, Chen-Yu Wang, Fei-Yuan Hsiao

**Affiliations:** 1 Division of Hematology, Department of Internal Medicine, National Taiwan University Hospital, Taipei, Taiwan; 2 Department of Hematological Oncology, National Taiwan University Cancer Center, Taipei, Taiwan; 3 Health Data Research Center, National Taiwan University, Taipei, Taiwan; 4 School of Pharmacy, National Taiwan University, Taipei, Taiwan; 5 Graduate Institute of Clinical Pharmacy, College of Medicine, National Taiwan University, 33, Linsen S. Rd, Taipei, 10050 Taiwan; 6 Department of Pharmacy, National Taiwan University Hospital, Taipei, Taiwan

**Keywords:** Diffuse large B cell lymphoma, Rituximab, Anthracycline, Taiwan Cancer registry database, Extremely elderly

## Abstract

**Background:**

The standard frontline therapy for patients with diffuse large B cell lymphoma (DLBCL) is R-CHOP. However, patients older than 80 years are excluded from clinical trials. The importance of rituximab and anthracycline remains unknown in extremely elderly DLBCL patients. Here, we incorporated data from the Taiwan Cancer Registry Database (TCRD), National Health Insurance Research Database (NHIRD), and National Death Registry to evaluate the clinical benefits of rituximab and anthracycline in elderly patients. From the TCRD and NHIRD, we included DLBCL patients aged older than 60 years who received R-CHOP, R-CVP, CHOP, or CVP between 2010 and 2015.

**Results:**

Of the 3228 eligible patients, 2559 were between 60 and 79 years (the 60–79 group), and 669 were older than 80 years (the 80+ group). The proportions of patients in the different Ann Arbor stages and the practice settings were similar in both groups. The male-to-female ratio and the Charlson comorbidity index (CCI) scores in the 80+ group were higher than those in the 60–79 group. Patients in the 60–79 group received R-CHOP more frequently than those in the 80+ group. In the 60–79 group, the median age of the patients receiving R-CVP or CVP was older than those receiving R-CHOP or CHOP. In the analysis of overall survival (OS) and time to treatment failure (TTF), R-CHOP, female sex, younger age, lower Ann Arbor stage, lower CCI score, and care at a medical center predicted a favorable prognosis in the 60–79 group. However, only R-CHOP, younger age, and lower Ann Arbor stage remained independent favorable prognostic factors in the 80+ group.

**Conclusions:**

Our population-based study demonstrated the clinical benefits of rituximab and anthracycline in extremely elderly Asian patients with DLBCL.

## Background

Diffuse large B cell lymphoma (DLBCL) is the most common type of non-Hodgkin lymphoma (NHL) in both Western countries and Asia [1]. The standard treatment for newly diagnosed DLBCL patients is R-CHOP, which has been established by two prospective randomized trials, the Groupe d’Etude des Lymphomes de l’Adulte (GELA) non-Hodgkin lymphoma trial (GELA LNH-98.5) [2, 3], and the subsequent RICOVER-60 trial [4]. The median age at diagnosis for patients with DLBCL is more than 60 years [1]. Therefore, both trials included patients aged between 60 and 80 years. This raises an important issue in the frontline treatment of extremely old DLBCL patients, especially with regard to those older than 80 years. The clinical benefits of rituximab and anthracycline in newly diagnosed and extremely old DLBCL patients are unknown.

The two prospective randomized clinical trials, the GELA LNH-98.5 and RICOVER-60 trials, found that the addition of rituximab to CHOP substantially improves the prognosis of DLBCL patients [2, 4]. The long-term follow-up in the GELA LNH-98.5 trial also demonstrated the long-term benefit of rituximab in DLBCL patients [3]. However, both trials only included patients aged between 60 and 80 years [2, 4]. Although R-miniCHOP, a dose reduced regimen of R-CHOP, has been reported to be a safe and effective treatments for elderly DLBCL patients in a phase II study [5], the additional benefit of rituximab is not demonstrated in this trial becasue of its single-arm study design. Similar limitation was found in one retrospective analysis from a multicenter study. Although this retrospective analysis showed that R-CHOP or R-CHOP like regimens improve the overall survival in patients aged 80 years and older with DLBCL or grade 3B follicular lymphoma, their reference group was palliative care, which failed to demonstrate the additional benefit of rituximab [6]. Because rituximab may increase the incidence of opportunistic infections, [7] the benefits associated with the addition of rituximab remain debatable in the treatment of DLBCL patients older than 80 years. To the best of our knowledge, there is no population-based study examining the additional benefit of rituximab in DLBCL patients aged 80 years and older.

Another important issue in DLBCL patients is the use of anthracycline, which has cardiac toxicity and is contraindicated for patients with cardiac dysfunction. Most studies regarding the importance of anthracycline in DLBCL were retrospective and only included patients aged between 60 and 80 years old [8]. Therefore, the role of anthracycline remains debatable in DLBCL patients older than 80 years. Three retrospective studies showed that adding anthracycline did not influence overall survival in DLBCL patients older than 80 years [6, 9, 10]. However, one retrospective study and two population-based analyses from the Surveillance, Epidemiology, and End Results (SEER)-Medicare data set reported that anthracycline-containing regimens improved OS compared with anthracycline-free regimens [11–13]. However, there is a lack of information about the importance of anthracycline in Asian patients with DLBCL, especially in extremely elderly patients.

In our study, we investigated the importance of rituximab and anthracycline in elderly DLBCL patients in the real-world and population settings. We used data from the Taiwan Cancer Registry Database (TCRD), which included more than 90% of the cancer patients in Taiwan with histological confirmation. We incorporated the data from the TCRD with Taiwan’s National Health Insurance Research Database (NHIRD) and the National Death Registry for further analysis.

## Results

### Use of rituximab and anthracycline in older patients

The median age of DLBCL patients at the time of diagnosis in Taiwan was approximately 63 to 65 years [1]. Therefore, the patients aged between 60 and 79 years (the 60–79 group) accounted for the majority of DLBCL patients. To illustrate the differences in clinical practice regarding patients older than 80 years (the 80+ group), we compared the patients in the 60–79 group to those in the 80+ group.

The distributions of patients in the different Ann Arbor stages at diagnosis and the proportions of patients treated at medical centers were similar in both groups (Table 1). However, the male-to-female ratio was higher in the 80+ group than in the 60–79 group (1.06:1 for the 60–79 group and 1.82:1 for the 80+ group; *P* < 0.0001; Table 1). The CCI scores were also higher in the 80+ group than in the 60–79 group (*P* < 0.0001; Table 1). The patients in the 80+ group tended to have more comorbidities such as congestive heart failure, cerebrovascular diseases, dementia, chronic pulmonary diseases and renal diseases (Supplementary Table 1), whereas the patients in the 60–79 group had more mild liver diseases (Supplementary Table 1).

**Table 1:** Patient characteristics

	Aged between 60 and 79 years										Aged more than 80 years									
	Total		R-CHOP		R-CVP		CHOP		CVP		Total		R-CHOP		R-CVP		CHOP		CVP	
	n	(%)	n	(%)	n	(%)	n	(%)	n	(%)	n	(%)	n	(%)	n	(%)	n	(%)	n	(%)
**Patient number**	2559		1838		539		119		63		669		258		335		23		53	
**Mean of Age (SD)**	69.1	(5.73)	68.0	(5.49)	72.5	(5.10)	69.2	(5.76)	72.4	(5.23)	84.0	(3.39)	83.2	(2.96)	84.6	(3.54)	84.4	(3.71)	84.5	(3.48)
**Gender**																				
**Male**	1318	(51.5)	972	(52.9)	248	(46.0)	69	(58.0)	29	(46.0)	432	(64.6)	186	(72.1)	198	(59.1)	15	(65.2)	33	(62.3)
**Female**	1241	(48.5)	866	(47.1)	291	(54.0)	50	(42.0)	34	(54.0)	237	(35.4)	72	(27.9)	137	(40.9)	8	(34.8)	20	(37.7)
**Ann Arbor stage**																				
**I**	422	(16.5)	309	(16.8)	87	(16.1)	17	(14.3)	9	(14.3)	109	(16.3)	43	(16.7)	58	(17.3)	4	(17.4)	4	(7.5)
**II**	658	(25.7)	488	(26.6)	129	(23.9)	31	(26.1)	10	(15.9)	150	(22.4)	59	(22.9)	77	(23.0)	5	(21.7)	9	(17.0)
**III**	610	(23.8)	437	(23.8)	140	(26.0)	23	(19.3)	10	(15.9)	182	(27.2)	70	(27.1)	92	(27.5)	6	(26.1)	14	(26.4)
**IV**	869	(34.0)	604	(32.9)	183	(34.0)	48	(40.3)	34	(54.0)	228	(34.1)	86	(33.3)	108	(32.2)	8	(34.8)	26	(49.1)
**Practice setting**																				
**medical center**	1697	(66.3)	1232	(67.0)	350	(64.9)	80	(67.2)	35	(55.6)	452	(67.6)	174	(67.4)	233	(69.6)	12	(52.2)	33	(62.3)
**others**	862	(33.7)	606	(33.0)	189	(35.1)	39	(32.8)	28	(44.4)	217	(32.4)	84	(32.6)	102	(30.4)	11	(47.8)	20	(37.7)
**Charlson comorbidity index**																				
**0**	931	(36.4)	712	(38.7)	158	(29.3)	43	(36.1)	18	(28.6)	191	(28.6)	84	(32.6)	86	(25.7)	7	(30.4)	14	(26.4)
**1**	788	(30.8)	571	(31.1)	162	(30.1)	37	(31.1)	18	(28.6)	203	(30.3)	74	(28.7)	107	(31.9)	4	(17.4)	18	(34.0)
**2+**	840	(32.8)	555	(30.2)	219	(40.6)	39	(32.8)	27	(42.9)	275	(41.1)	100	(38.8)	142	(42.4)	12	(52.2)	21	(39.6)

Regarding the treatment selection, the majority of the patients in the 60–79 group received R-CHOP (71.8%), but the majority in the 80+ group received R-CVP (50.1%; Table 1). Regarding the use of rituximab, fewer patients in the 80+ group (88.6%) than in the 60–79 group received rituximab (92.9%; *P* = 0.0003; Table 1). The proportion of those receiving additional anthracycline was also lower in the 80+ group (42.0%) than in the 60–79 group (76.5%; *P* < 0.0001; Table 1).

The median age at diagnosis in the 80+ group was 84.0 years and that in the 60–79 group was 69.1 years (Table 1). However, in the 60–79 group, the median age of the patients receiving R-CVP and CVP was older than those receiving R-CHOP and CHOP (median age, 68.0 years for R-CHOP and 69.2 years for CHOP, versus 72.5 years for R-CVP and 72.4 years for CVP; Table 1).

### R-CHOP improved overall survival and time to treatment failure cross-generationally

We included all patients with newly diagnosed DLBCL between 2010 and 2015 in the survival analysis and showed that the OS decreased with the increasing age of the patients (Supplementary Fig. 2). The median OS for the DLBCL patients aged between 60 and 69 years was not reached, that for those aged between 70 and 79 years was 34.52 months and that for those older than 80 years was 11.84 months (Supplementary Fig. 2).

**Fig. 1:**
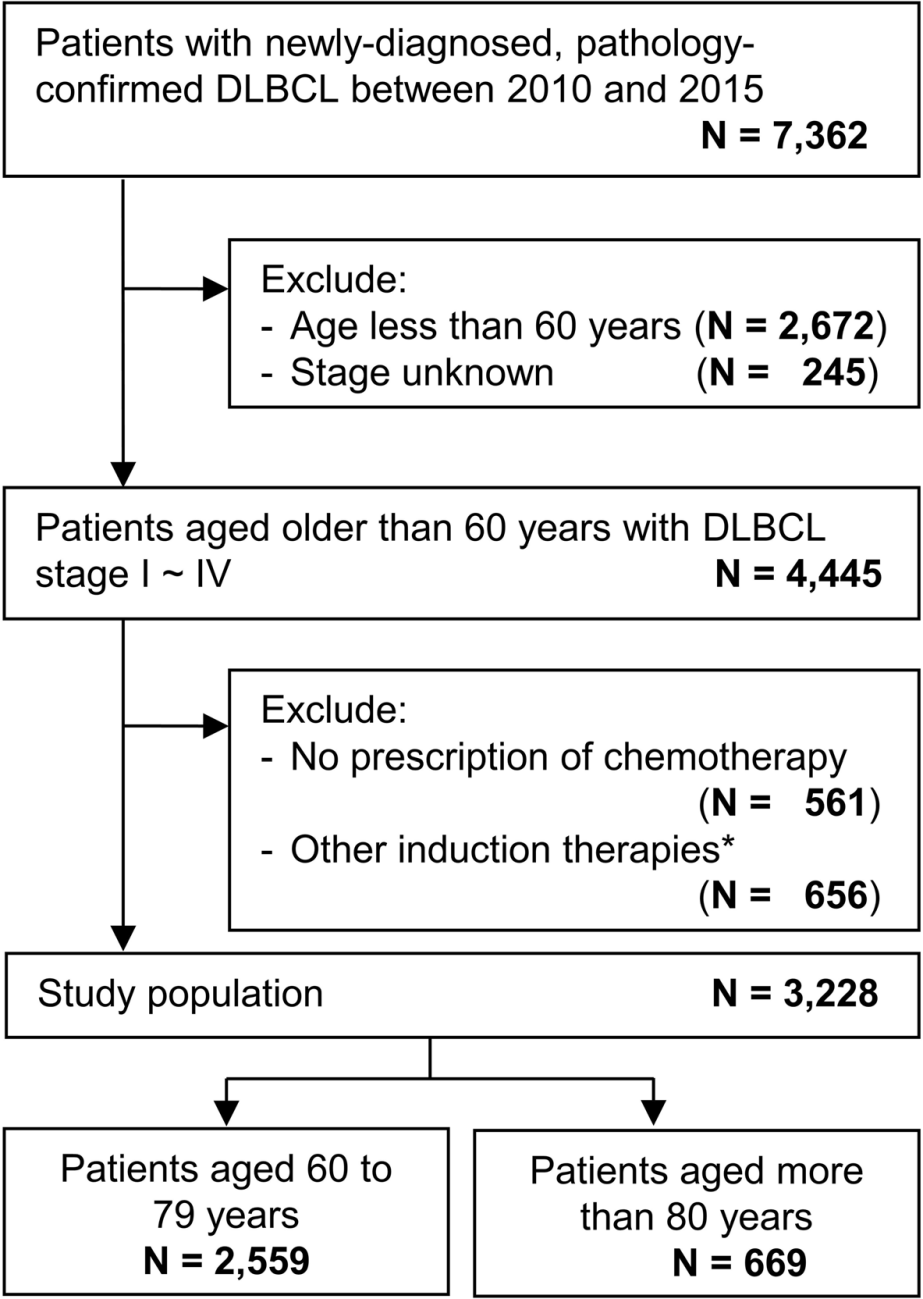
Algorithm of the study cohort selection. DLBCL, diffuse large B cell lymphoma. *Chemotherapies other than R-CHOP, R-CVP, CHOP and CVP

**Fig. 2:**
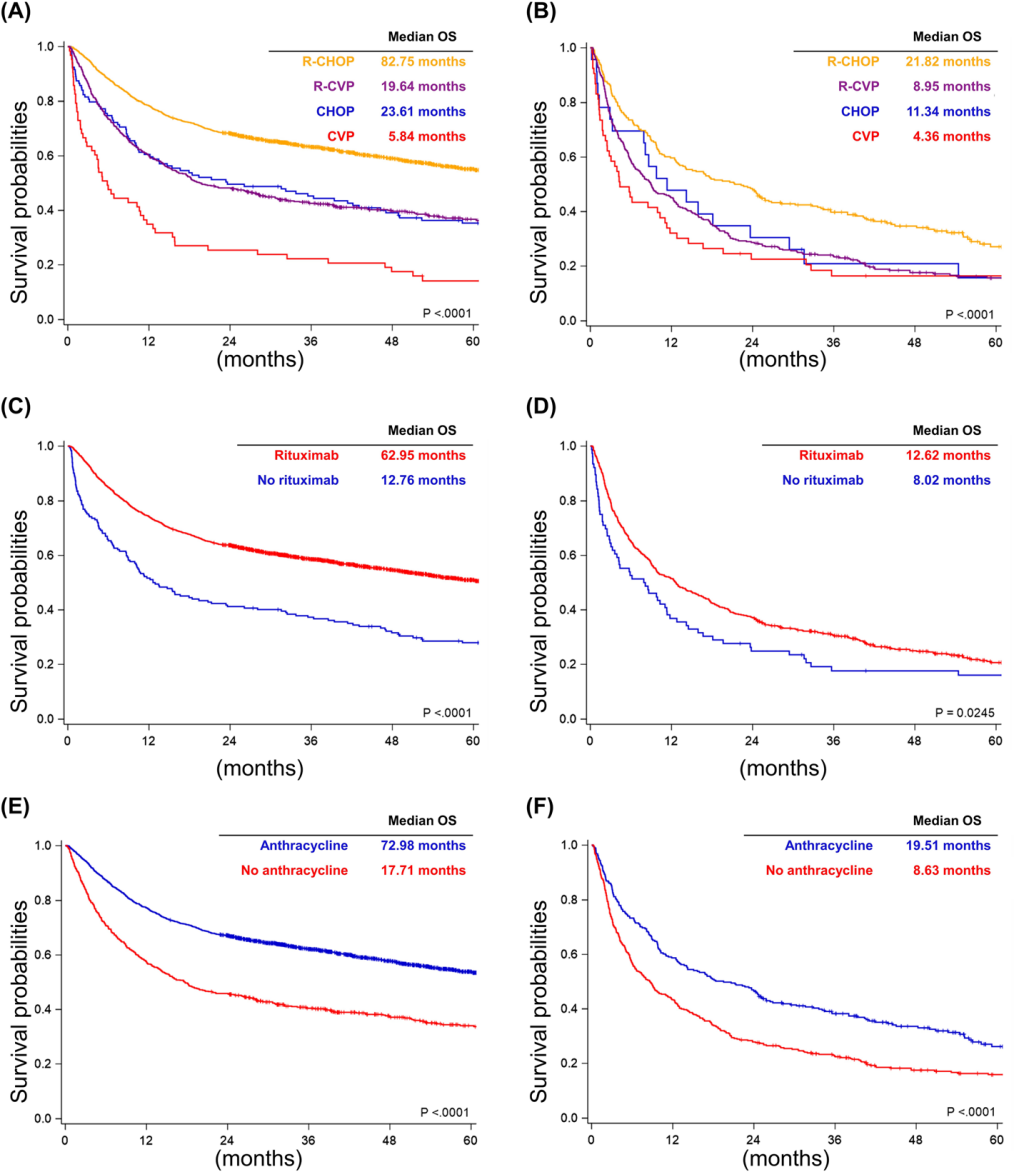
Overall survival. **a** Patients stratified by the frontline therapies in those aged between 60 and 79 years. **b** Patients stratified by the frontline therapies in those older than 80 years. **c** Patients stratified by the use of rituximab in those aged between 60 and 79 years. **d** Patients stratified by the use of rituximab in those older than 80 years. **e** Patients stratified by the use of anthracycline in those aged between 60 and 79 years. **f** Patients stratified by the use of anthracycline in those older than 80 years

To investigate the clinical influence of rituximab and anthracycline, we only included the patients receiving R-CHOP, R-CVP, CHOP and CVP for further analysis and stratified the patients according to their age (Fig. 1). The median follow-up duration was 35.6 months in the 60–79 group and 22.1 months in the 80+ group. In the analysis of OS, R-CHOP remained the best frontline treatment for both groups (median OS, 82.76 months in the 60–79 group, and 21.82 months in the 80+ group; Fig. 2a and b). In the 60–79 group, the OS times of the patients receiving R-CVP and CHOP were similar (median OS, 19.64 months for R-CVP and 23.61 months for CHOP; Fig. 2a), and the OS of those receiving CVP was obviously poor compared with the others (5.84 months; Fig. 2a). In the 80+ group, the OS times of the patients receiving R-CVP, CHOP, and CVP were similar (median OS, 8.95 months for R-CVP, 11.34 months for CHOP, and 4.36 months for CVP; Fig. 2b). Only R-CHOP significantly improved the OS in the 80+ group (median OS, 21.82 months**;** Fig. 2b). When we stratified the patients according to the use of rituximab and anthracycline, the use of rituximab and anthracycline improved OS in both groups (Fig. 2c, d, e, and f).

On the other hand, the importance of CCI scores was also different in both age groups (Supplementary Fig. 3). In the 60–79 group, the OS was shorter if the patients had higher CCI scores (Supplementary Fig. 3A). In the 80+ groups, CCI scores did not influence OS (Supplementary Fig. 3B). Further, we performed a subgroup analysis to clarify the effectiveness of R-CHOP in different age and CCI groups (Supplementary Fig. 4). In the 60–79 group, R-CHOP improved the OS no matter what CCI score the patients had (Supplementary Fig. 4A, 4C, 4E, and 4G). However, in the 80+ group, only the patients with CCI score 0 would benefit from the use of R-CHOP (Supplementary Fig. 4B).

The multivariate analysis showed that the application of R-CHOP, female sex, younger age, lower Ann Arbor stage, no radiotherapy, lower CCI score, and treatment in a medical center were favorable prognostic factors for OS in the 60–79 group (Table 2). However, sex, radiotherapy, CCI score, and the practice setting did not influence OS in the 80+ group (Table 2). Only the application of R-CHOP, younger age, and lower Ann Arbor stage remained independent favorable prognostic factors for OS in the 80+ group (Table 2).

**Table 2:** Adjusted hazard ratios of overall survival

Variable	Aged between 60 and 79 years			Aged more than 80 years		
	HR	(95%CI)	*p*-value	HR	(95%CI)	*p*-value
**Frontline treatment**						
R-CHOP	1		< 0.0001	1		0.0003
R-CVP	1.65	(1.44–1.89)		1.48	(1.22–1.80)	
CHOP	1.81	(1.44–2.28)		1.37	(0.85–2.21)	
CVP	3.08	(2.33–4.08)		1.65	(1.19–2.29)	
**Gender**						
male	1		0.0004	1		0.5606
female	0.82	(0.73–0.91)		0.95	(0.79–1.14)	
**Age**	1.03	(1.02–1.05)	< 0.0001	1.05	(1.02–1.07)	0.0001
**Ann Arbor stage**						
I	1		< 0.0001	1		< 0.0001
II	1.32	(1.07–1.62)		1.24	(0.92–1.68)	
III	2.07	(1.69–2.55)		1.53	(1.14–2.04)	
IV	3.06	(2.52–3.70)		2.19	(1.66–2.90)	
**Radiotherapy**						
No	1		0.0067	1		0.5483
Yes	1.21	(1.06–1.39)		1.07	(0.85–1.35)	
**Carlson comorbidity index**						
0	1		< 0.0001	1		0.7487
1	1.09	(0.95–1.26)		0.94	(0.75–1.18)	
2+	1.43	(1.25–1.64)		1.02	(0.82–1.26)	
**Practice setting**						
medical center	1		0.0130	1		0.5251
others	1.16	(1.03–1.30)		1.06	(0.88–1.28)	

**Fig. 3:**
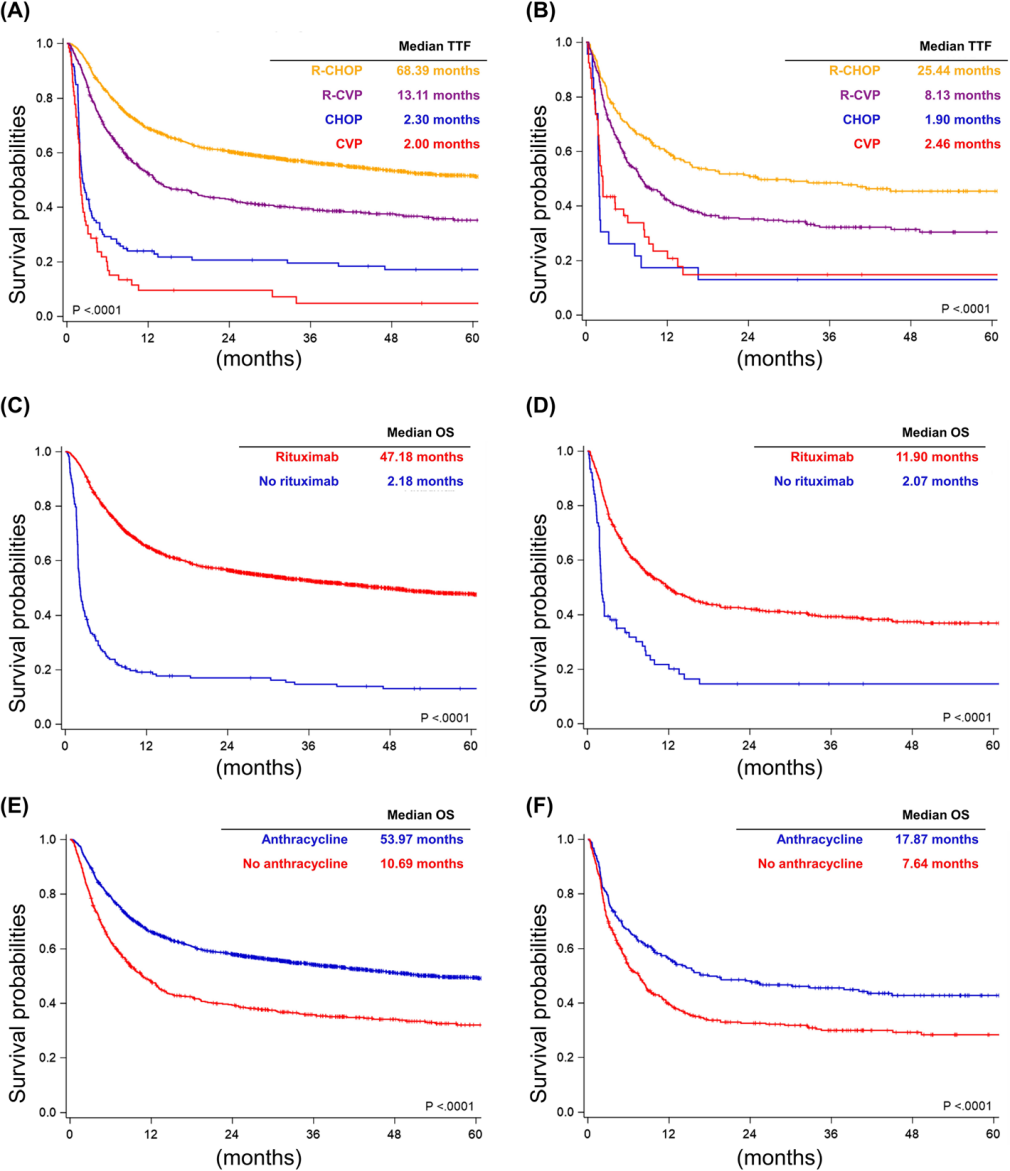
Time to treatment failure. **a** Patients stratified by the frontline therapies in those aged between 60 and 79 years. **b** Patients stratified by the frontline therapies in those older than 80 years. **c** Patients stratified by the use of rituximab in those aged between 60 and 79 years. **d** Patients stratified by the use of rituximab in those older than 80 years. **e** Patients stratified by the use of anthracycline in those aged between 60 and 79 years. **f** Patients stratified by the use of anthracycline in those older than 80 years

In the analysis of TTF, R-CHOP remained the best frontline treatment for both groups (median TTF, 68.39 months in the 60–79 group and 25.44 months in the 80+ group; Fig. 3a and b). The subgroup analysis also showed that the use of rituximab and anthracycline improved TTF in both groups (Fig. 3c, d, e, and f). In the 60–79 group, the use of R-CHOP, female sex, lower Ann Arbor stage and lower CCI score were independent favorable prognostic factors for TTF (Table 3). However, only the use of R-CHOP and lower Ann Arbor stage remained independent favorable prognostic factors of TTF in the 80+ group (Table 3).

**Table 3:** Adjusted hazard ratios of time to treatment failure

Variable	Aged between 60 and 79 years			Aged more than 80 years		
	HR	(95%CI)	*p*-value	HR	(95%CI)	*p*-value
**Frontline treatment**						
R-CHOP	1		< 0.0001	1		< 0.0001
R-CVP	1.64	(1.43–1.88)		1.52	(1.22–1.91)	
CHOP	4.49	(3.63–5.57)		3.58	(2.21–5.81)	
CVP	6.70	(5.08–8.82)		3.00	(2.11–4.29)	
**Gender**						
male	1		0.0271	1		0.4817
female	0.88	(0.79–0.99)		0.93	(0.75–1.14)	
**Age**	1.01	(1.00–1.02)	0.1303	1.03	(1.00–1.05)	0.0915
**Ann Arbor stage**						
I	1		< 0.0001	1		< 0.0001
II	1.40	(1.14–1.72)		1.22	(0.85–1.74)	
III	2.24	(1.83–2.74)		1.57	(1.12–2.20)	
IV	3.12	(2.58–3.77)		2.24	(1.62–3.09)	
**Radiotherapy**						
No	1		0.6740	1		0.1561
Yes	1.03	(0.90–1.19)		0.82	(0.62–1.08)	
**Carlson comorbidity index**						
0	1		0.0008	1		0.7690
1	1.07	(0.93–1.23)		1.03	(0.80–1.34)	
2+	1.27	(1.12–1.45)		1.09	(0.85–1.40)	
**Practice setting**						
medical center	1		0.3868	1		0.5453
others	1.05	(0.94–1.18)		0.94	(0.76–1.16)	

## Discussion

Our study is the first population-based study to investigate the use of rituximab and anthracycline in extremely elderly patients with DLBCL, especially in the Asian population. We compared the extremely elderly patients with the majority of patients aged between 60 and 79 years. We showed that adding rituximab or anthracycline not only improved the prognosis in the majority of DLBCL patients aged between 60 and 79 years but also in the extremely elderly patients. These findings provide real-world evidence supporting the benefits of rituximab and anthracycline.

Regarding the addition of rituximab, we included DLBCL patients aged between 60 and 79 years, which was compatible with the patients included in the GELA LNH-98.5 trial and the RICOVER-60 trial [2–4]. Our study showed that the survival benefit of rituximab could be translated from clinical trials into real-world practice (Fig. 2c and 3c). Furthermore, we also included DLBCL patients older than 80 years who received R-CHOP, R-CVP, CHOP, or CVP. Patients older than 80 years were excluded from most clinical trials. The clinical benefit of rituximab in extremely elderly patients remained uncertain because rituximab also increased the risk of infections [7]. Here, we demonstrated that rituximab improved the OS and TTF in extremely elderly patients (Fig. 2d and 3d). Therefore, our population-based study illustrated that the clinical benefit of rituximab could be extended from the majority population (including those aged between 60 and 79 years) to the extremely elderly population (aged more than 80 years) in the real-world practice setting.

Adding anthracycline to the frontline treatment of DLBCL patients has been investigated for decades, especially in the aged population. However, it is unethical to conduct a clinical trial to answer the question. Real-world evidence provides a good opportunity to fill this gap. The results from several retrospective studies were inconsistent. A retrospective study from four institutions showed that the addition of anthracycline improved the outcome in DLBCL patients older than 80 years [14]. Another retrospective study from the Veteran’s Health Administration cancer registry in the United States showed that anthracycline did not influence mortality in DLBCL patients older than 80 years [9]. The study from the GELTAMO Spanish Collaborative Group reported that the survival among patients receiving R-CHOP, R-CHOPr (R-CHOP with any type of dose reduction), and R-CVP were similar [6]. Another retrospective cohort in Japan also showed that the treatment outcome of R-CVP was similar to that of R-CHOP [10]. However, a retrospective study from the MD Anderson Cancer Center reported that R-CHOP or R-EPOCH improved the outcome in very elderly patients with DLBCL compared with R-CVP [11]. Because of the inconsistency, two population-based analyses from the SEER-Medicare database also attempted to clarify the outcome in extremely elderly patients with DLBCL [12, 13]. Both reported that R-CHOP improved the OS compared with the anthracycline-free regimens [12, 13]. However, there is still limited data about the influence of anthracycline in the Asian population. Here, we conducted the first Asian population-based study and demonstrated that the addition of anthracycline retained a survival benefit in extremely elderly DLBCL patients. Noteworthy, this survival benefit of adding anthracycline may be limited to those with CCI scored to 0 in DLBCL patients aged 80 and older. (Fig. 2f and 3f).

However, there were several limitations of our study. First, the body surface area was not available in our claim-based database, and we could not tell the difference between R-CHOP and R-miniCHOP. Some patients receiving R-CHOP in our study might receive R-miniCHOP indeed. Second, in our claim-based database, serum LDH levels were also not included, and the information of the extra-nodal involvement and performance score was not comprehensive. We were not able to calculate the international prognostic index (IPI). However, we included the Ann Arbor stage and age in the multivariate analysis (Table 2 and Table 3), which were part of the IPI score. The multivariate analysis still demonstrated the survival benefit of additional rituximab and anthracycline in patients aged 80 years and older. Third, the side effects of chemotherapies were not available in our claim-based database. It was difficult to know whether the therapy-related mortality rate increased in the high-intensity chemotherapy groups or not. Fourth, our study was a retrospective population-based design. The patient characteristics were not equally distributed in each treatment arm. In the real-world setting, physicians tended to avoid anthracycline in elderly patients or those with cardiac diseases. Therefore, the patients receiving R-CVP or CVP were older and had higher CCI scores. However, in the multivariate analysis, our study still indicated that the patients would have a better prognosis if they were eligible to receive rituximab or anthracycline.

## Conclusions

Our study is the first Asian population-based study to illustrate the clinical benefit of rituximab in extremely elderly DLBCL patients. Furthermore, our study is also the first Asian population-based study of this issue, which shows that additional anthracycline improves survival in extremely elderly DLBCL patients.

## Methods

### Data source

We retrospectively included patients with DLBCL diagnosed during 2010 to 2015 from the TCRD. Furthermore, we incorporated the information from Taiwan’s NHIRD regarding the usage of chemotherapies and rituximab and the survival data from the National Death Registry until December 31, 2017 (Supplementary Fig. 1). The TCRD is a government-funded program launched by the Ministry of Health and Welfare (MOHW) in Taiwan. The TCRD was initiated in 1979. The data integrity of the TCRD has dramatically improved since the Cancer Control Act was introduced in 2003. In the TCRD, 91.5% of the patients had morphological verification of the cancer diagnosis, and 98.4% had comprehensive clinical information. Therefore, the TCRD is an informative nationwide database for cancer surveillance in Taiwan [15, 16]. The NHIRD is a nationwide claim-based database comprising more than 99% of health care utilizations in Taiwan [15]. The National Death Registry contains comprehensive survival data for the Taiwanese population. The encrypted and unique identification numbers of the insured, which were assigned to ensure confidentiality, were interconnected among all database subsets contained in Taiwan’s mandatory National Health Insurance system. The protocol of our study was approved by the Research Ethics Committee of National Taiwan University Hospital (registration number, 201604051 W)**.**

### Study population

All cancer types have been coded in the TCRD based on the International Classification of Diseases for Oncology, the Third Edition (ICD-O-3) since 2002 [16]. DLBCL in this study was defined through the ICD-O-3 code along with the 2008 WHO classification of lymphoid neoplasms (ICD-O-3 codes for DLBCL in this study, 96,803, 96,843, 96,883, 97,123, 97,373, 97,353, and 97,383; Supplementary Table 2) [17].

The processes used to identify the study population were illustrated in Fig. 1. Between 2010 and 2015, 7362 patients in Taiwan were diagnosed with DLBCL. We excluded patients younger than 60 years (*n* = 2672), those with unknown stage (*n* = 245), and those who did not receive any chemotherapy (*n* = 561) or chemotherapies other than R-CHOP, R-CVP, CHOP, and CVP (*n* = 656) as frontline treatments (Fig. 1). Three thousand two hundred and twenty eight patients were enrolled for further analysis, including 2559 between 60 and 79 years (the 60–79 group) and 669 older than 80 years (the 80+ group; Fig. 1). The follow-up period for eligible patients was from the start of frontline treatments to the date of death or December 31, 2017, whichever occurred first.

### Definition of treatments

Anatomical Therapeutic Chemical (ATC) codes [18] were applied to extract rituximab (L01XC02), cyclophosphamide (L01AA01), doxorubicin (L01DB01), daunorubicin (L01DB02), epirubicin (L01DB03), aclarubicin (L01DB04), zorubicin (L01DB05), idarubicin (L01DB06), mitoxantrone (L01DB07), vincristine (L01CA02), prednisone (H02AB), and all other possible antineoplastic and immune-modulating agents that might be prescribed to patients with DLBCL from Taiwan’s NHIRD. A change of regimens was defined as any adjustment of a chemotherapy regimen except regimen switching between CHOP and CVP or between R-CHOP and R-CVP. Rituximab was counted as part of the frontline treatment if the interval between the start date of rituximab and that of other chemotherapy agents was less than 42 days. If the patients did not receive chemotherapy for more than 3 months, it was counted as a cessation of chemotherapy. If they started any chemotherapy later, we classified it as a second-line treatment whether the regimen was the same as the frontline treatment or not. For radiotherapy, the specific payment codes were 36006B, 36009B, 36010B, 36011B, 36012B, 36013B, 36018B, 36019B or 37010B (Supplementary Table 3). If radiotherapy was initiated within 3 months after the end of frontline treatment, the radiotherapy was counted as part of the frontline treatments.

### Endpoints

The primary endpoint of this study was overall survival (OS), which was defined as the duration between the index date (the start date of the frontline treatments) and the date of death. If the patients were still alive at the end of 2017, they were censored. The secondary endpoint was the time to treatment failure (TTF). Refractory was defined as the need to receive other intravenous chemotherapy different from the frontline chemotherapy within 3 months after the end of the frontline treatments. Relapse was defined as the need to receive any intravenous chemotherapy more than 3 months after the end of the frontline treatments. The duration of TTF was between the index date and the date of relapse, refractory, or death within 3 months after the end of frontline treatments. The patients were censored if the duration between the death date and the end of the frontline treatments was more than 3 months or if they did not experience relapse, refractory or death by the end of 2017.

### Statistical analysis

All data were analyzed with SAS® software, version 9.4 (SAS Institute, Cary, NC, USA). Student’s t-tests were applied to compare continuous variables, and X^2^ tests were used for categorical variables. Cox proportional hazard models were performed to determine whether there were significant differences in OS and TTF according to several categorical variables. To further control for potential confounding factors, all models were adjusted for sex (grouped as male and female), Ann Arbor stage of DLBCL (grouped as stage I, II, III, IV) [19], use of radiotherapy or not, Charlson comorbidity index score (CCI score; grouped as 0, 1, and 2+) [20–22], and the setting in which patients were primarily cared for (grouped as medical center and others). The adjusted hazard ratios (aHRs) were presented with a confidence interval stated at the 95% level. All statistical tests were two-sided, and the significance level was set at 0.05.

## Supplementary information

**Additional file 1: Supplementary Fig. 1** The illustration of study period. **Supplementary Fig. 2** Overall survival of the patient with DLBCL diagnosed between 2010 and 2015. NR, not reached. **Supplementary Fig. 3** Overall survival according to the Charlson comorbidity index score. (A) Patients aged between 60 and 79 years, (B) Patients older than 80 years. **Supplementary Fig. 4** Overall survival. Patients were stratified according to the type of induction chemotherapy in each group. (A) Patients aged between 60 and 79 years with CCI score 0, (B) Patients older than 80 years with CCI score 0, (C) Patients aged between 60 and 79 years with CCI score 1, (D) Patients older than 80 years with CCI score 1, (E) Patients aged between 60 and 79 years with CCI score 2, (F) Patients older than 80 years with CCI score 2, (G) Patients aged between 60 and 79 years with CCI score 3+, (H) Patients older than 80 years with CCI score 3+. **Supplementary Table 1** Details of Carlson comorbidity index. **Supplementary Table 2** The M-code of diagnosis in this study. **Supplementary Table 3** The payment code and name of radiotherapy.

## Data Availability

The datasets used and/or analyzed during the current study are available from the corresponding author on reasonable request.

## Abbreviations

CCI: Charlson comorbidity index
CHOP: Cyclophosphamide, doxorubicin, vincristine, prednisolone
CVP: Cyclophosphamide, doxorubicin, vincristine, prednisolone
DLBCL: Diffuse large B cell lymphoma
ICD-O-3: International Classification of Diseases for Oncology, the Third Edition
NHIRD: National Health Insurance Research Database
NHL: Non-Hodgkin lymphoma
OS: Overall survival
R-CHOP: Rituximab, cyclophosphamide, doxorubicin, vincristine, prednisolone
R-CVP: Rituximab, cyclophosphamide, vincristine, prednisolone
R-EPOCH: Rituximab, etoposide, prednisone, vincristine, cyclophosphamide, doxorubicin
SEER: Surveillance, Epidemiology, and End Results
TCRD: Taiwan Cancer Registry Database
TTF: Time to treatment failure
