# Supplementary material for: Frontline treatments in extremely elderly patients with diffuse large B-cell lymphoma: a population-based study in Taiwan, 2010–2015
Source: Immun Ageing. 2020 Jun 10;17:17. doi: 10.1186/s12979-020-00188-8 (PMC7285734; doi:10.1186/s12979-020-00188-8)
Supplement: Supplementary file 1 — Additional file 1: Supplementary Fig. 1 The illustration of study period. Supplementary Fig. 2 Overall survival of the patient with DLBCL diagnosed between 2010 and 2015. NR, not reached. Supplementary Fig. 3 Overall survival according to the Charlson comorbidity index score. (A) Patients aged between 60 and 79 years, (B) Patients older than 80 years. Supplementary Fig. 4 Overall survival. Patients were stratified according to the type of induction chemotherapy in each group. (A) Patients aged between 60 and 79 years with CCI score 0, (B) Patients older than 80 years with CCI score 0, (C) Patients aged between 60 and 79 years with CCI score 1, (D) Patients older than 80 years with CCI score 1, (E) Patients aged between 60 and 79 years with CCI score 2, (F) Patients older than 80 years with CCI score 2, (G) Patients aged between 60 and 79 years with CCI score 3+, (H) Patients older than 80 years with CCI score 3+. Supplementary Table 1 Details of Carlson comorbidity index. Supplementary Table 2 The M-code of diagnosis in this study. Supplementary Table 3 The payment code and name of radiotherapy. [file 12979_2020_188_MOESM1_ESM.pdf]

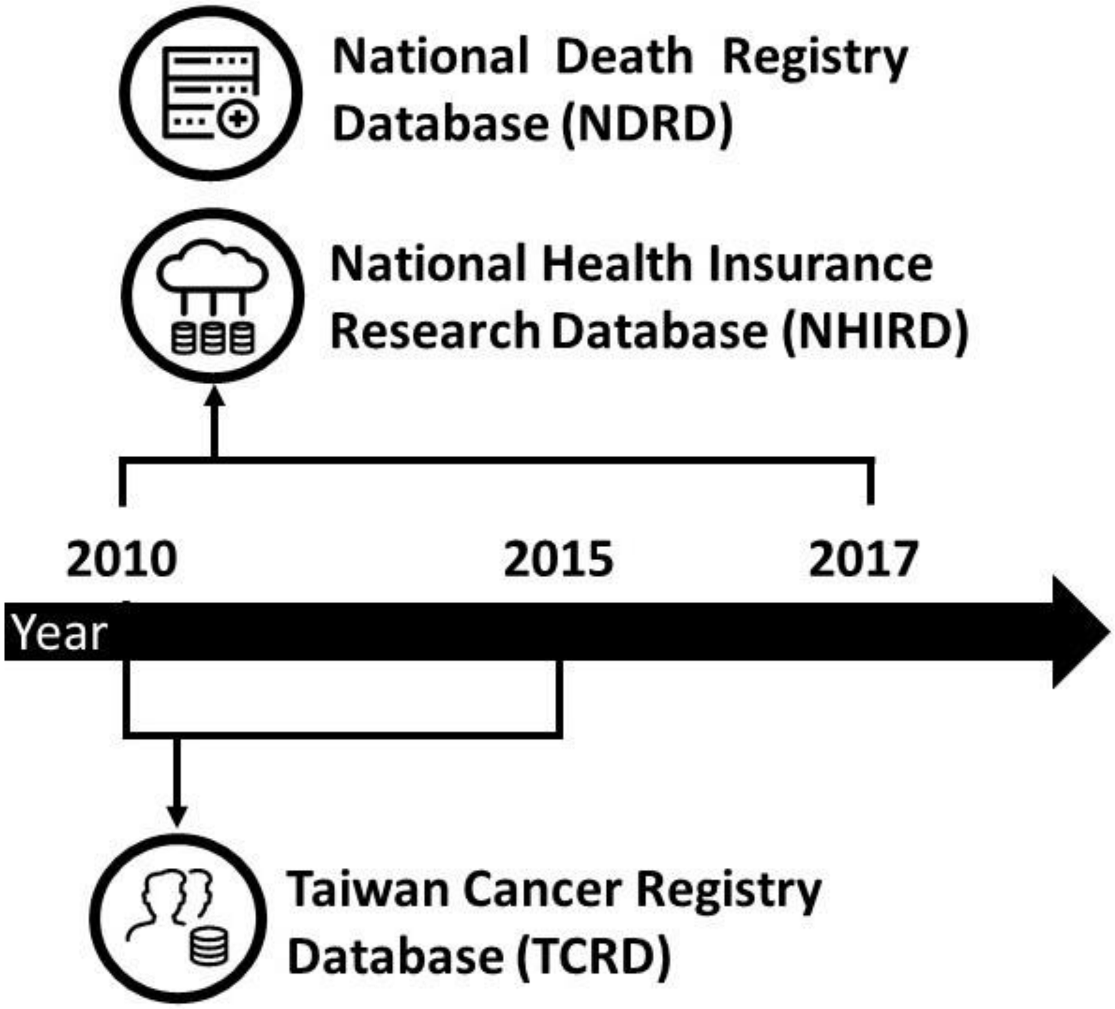

Supplementary Figure 1. The illustration of study period

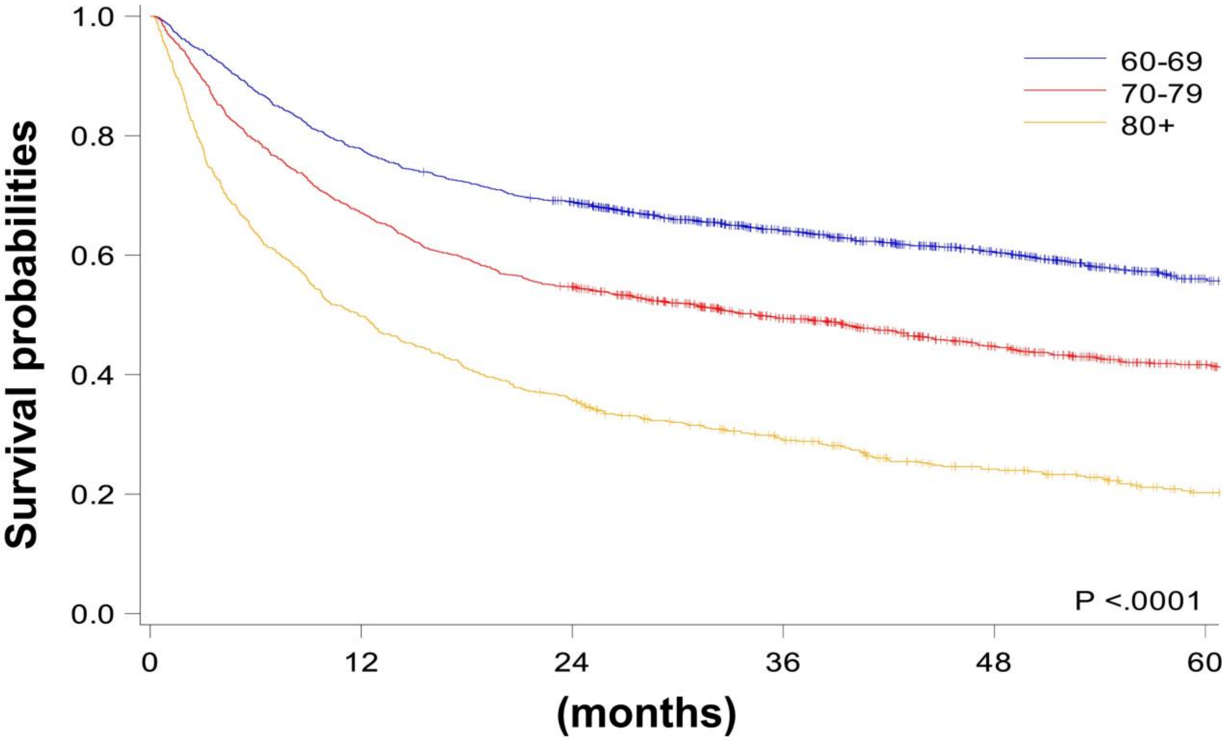

Age group	N	Survival Probabilities 1y	2y	3y	4y	5y	Survival Time (month) Median	95% CI for Median	P-value
60-69	1335	0.7775	0.6891	0.6404	0.6050	0.5605	NR	(76.56,.)	<0.0001
70-79	1224	0.6708	0.5474	0.4942	0.4467	0.4168	34.52	(27.80,42.49)	
80+	669	0.4978	0.3572	0.2901	0.2419	0.2024	11.84	(9.70,14.07)	

Supplementary Figure 2. Overall survival of the patient with DLBCL diagnosed between 2010 and 2015. NR, not reached.

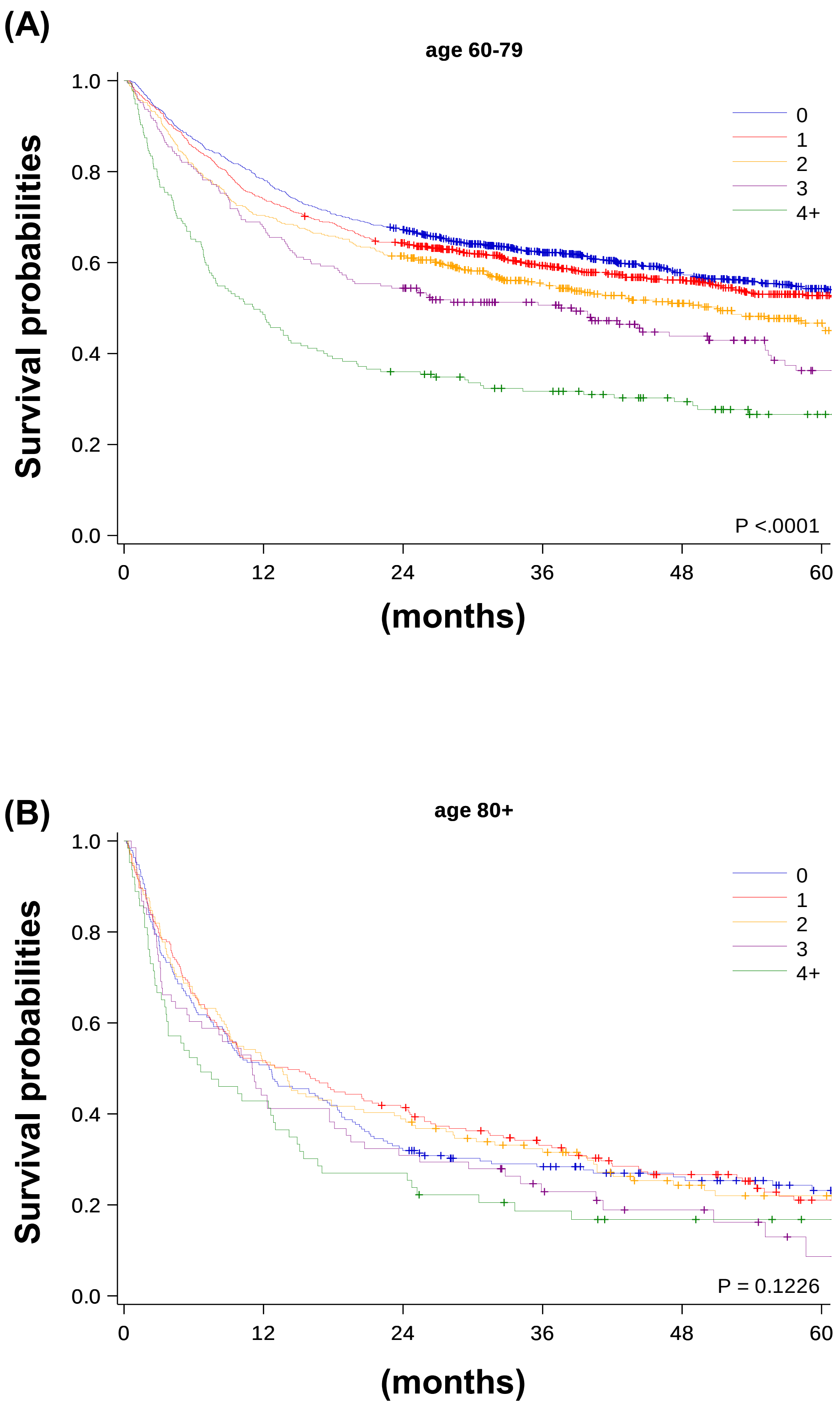

Supplementary Figure 3. Overall survival according to the Charlson comorbidity index score. **(A)** Patients aged between 60 and 79 years, **(B)** Patients older than 80 years.

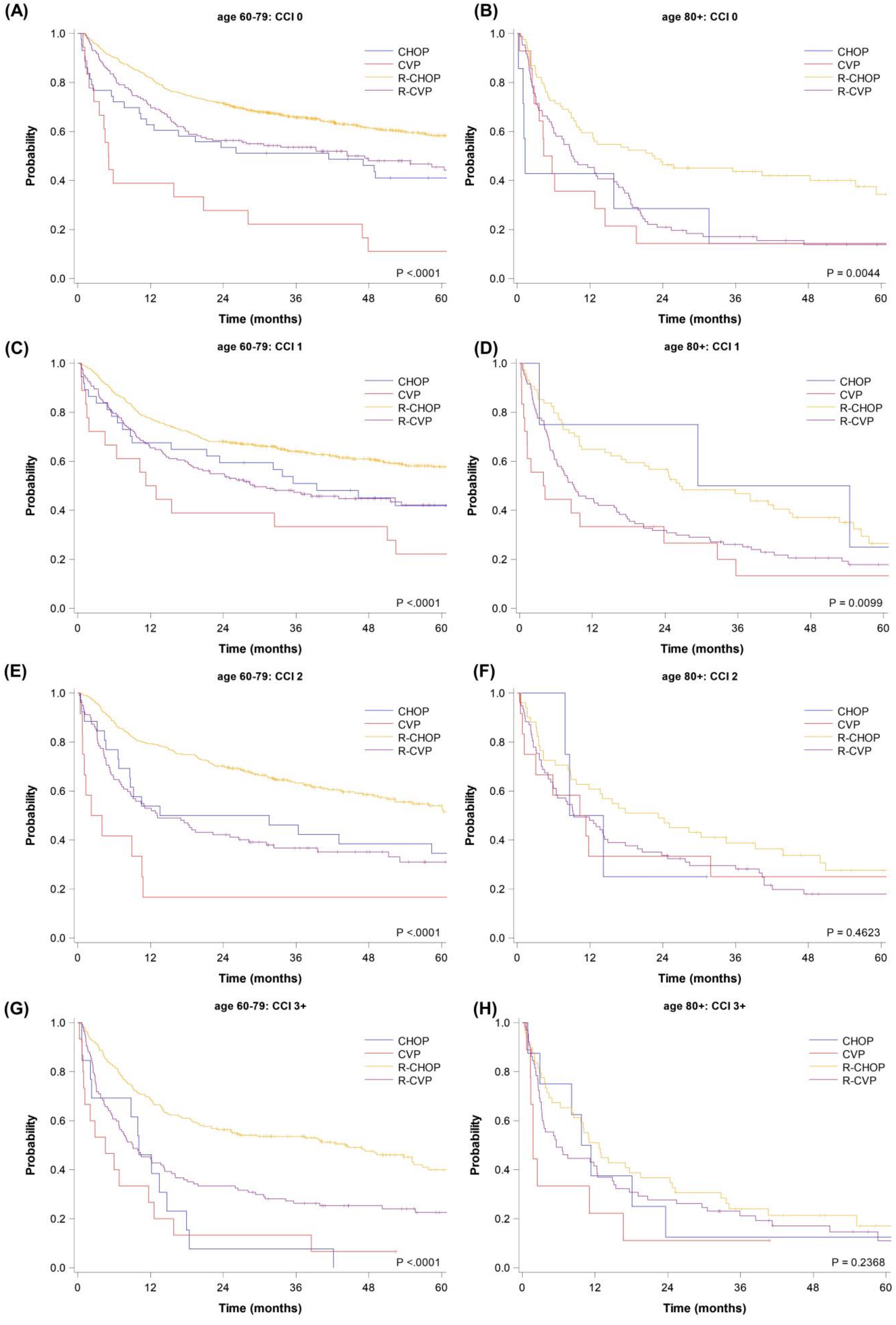

Supplementary Figure 4. Overall survival. Patients were stratified according to the type

of induction chemotherapy in each group. **(A)** Patients aged between 60 and 79 years with CCI score 0, **(B)** Patients older than 80 years with CCI score 0, **(C)** Patients aged between 60 and 79 years with CCI score 1, **(D)** Patients older than 80 years with CCI score 1, **(E)** Patients aged between 60 and 79 years with CCI score 2, **(F)** Patients older than 80 years with CCI score 2, **(G)** Patients aged between 60 and 79 years with CCI score 3+, **(H)** Patients older than 80 years with CCI score 3+.

Supplementary Table 1. Details of Carlson comorbidity index

Variable	All patients (N=3228) n	(%)	Aged between 60 and 79 years (N=2559) n	(%)	Aged more than 80 years (N=669) n	(%)	p-value
Myocardial infarction	28	(0.9)	22	(0.9)	6	(0.9)	0.9265
Congestive Heart failure	189	(5.9)	117	(4.6)	72	(10.8)	<0.0001
Peripheral vascular	70	(2.2)	55	(2.1)	15	(2.2)	0.8833
Cerebrovascular disease	358	(11.1)	252	(9.8)	106	(15.8)	<0.0001
Dementia	80	(2.5)	36	(1.4)	44	(6.6)	<0.0001
Chronic pulmonary disease	428	(13.3)	299	(11.7)	129	(19.3)	<0.0001
Rheumatic	119	(3.7)	98	(3.8)	21	(3.1)	0.3986
Peptic ulcer disease	759	(23.5)	590	(23.1)	169	(25.3)	0.2310
Mild liver disease	459	(14.2)	401	(15.7)	58	(8.7)	<0.0001
Moderate or severe liver disease	14	(0.4)	10	(0.4)	4	(0.6)	0.4679
Diabetes without chronic complication	776	(24.0)	625	(24.4)	151	(22.6)	0.3181
Diabetes with chronic complication	197	(6.1)	160	(6.3)	37	(5.5)	0.4874
Hemiplegia or paraplegia	29	(0.9)	26	(1.0)	3	(0.4)	0.1660
Renal disease	198	(6.1)	135	(5.3)	63	(9.4)	<0.0001

Supplementary Table 2. The M-code of diagnosis in this study

**M-code**	**ICD-O-3 Morphology**
96803	ML, large B-cell, diffuse
96843	ML, large B-cell, diffuse, immunoblastic, NOS
96883	T-cell histiocyte rich large B-cell lymphoma
97123	Intravascular large B-cell lymphoma
97373	ALK positive large B-cell lymphoma
97353	Plasmablastic lymphoma
97383	Lrg B-cell lymphoma in HHV8-assoc. multicentric Castleman DZ

Supplementary Table 3. The payment code and name of radiotherapy

Payment code	Name
36006B	obalt 60 teletherapy—each portal,simple
36009B	Cobalt 60 teletherapy, each portal,complicated
36010B	Emergent cobalt 60 teletherapy, each portal
36011B	Linear accelerator teletherapy,each simple portal
36012B	Linear accelerator teletherapy,each complex portal
36013B	Emergent linear accelerator teletherapy, each portal
36018B	Simulation procedure
36019B	Dosimetry
37010B	Interstital brachytherapy
